# The effect of probiotic supplementation during pregnancy on pregnancy complications: An umbrella meta-analysis

**DOI:** 10.1097/MD.0000000000046409

**Published:** 2025-12-19

**Authors:** Qian Gao, Yuanju Sun, Yuanyuan Qu, Fengling Li, Pan Li

**Affiliations:** a Department of Obstetrics and Gynecology, Yantai Affiliated Hospital of Binzhou Medical University, Yantai, China; b Department of Nephrology, Yantai Affiliated Hospital of Binzhou Medical University, Yantai, China.

**Keywords:** gestational diabetes, preeclampsia, pregnancy complications, probiotics, umbrella meta-analysis

## Abstract

**Background:**

Previous studies have suggested that probiotics might help prevent pregnancy complications, although recent meta-analyses have shown inconsistent results. To address this, an umbrella meta-analysis was conducted to investigate the impact of probiotics on pregnancy complications.

**Methods:**

PubMed and Scopus databases were systematically searched for relevant meta-analyses, spanning from their inception to April 2024. The primary outcomes of interest were risk of gestational diabetes (GDM) and preeclampsia, and the secondary outcomes were gestational weight gain, gestational age (GA) at delivery, and cesarean section. A random-effects model was utilized to combine relative risks (RR) and standardized mean differences along with their respective 95% confidence intervals (CI) for categorical and continuous outcomes, respectively.

**Results:**

In total, 27 studies involving 83,817 participants were incorporated. Probiotics supplementation notably decreased GDM risk (RR = 0.71, 95% CI: 0.61–0.83) while raised the odds of preeclampsia (RR = 1.23, 95% CI: 1.07–1.42) and increased GA (standardized mean differences = 0.07, 95% CI: 0.02 to 0.11). Subgroup analyses supported the preventive effect on GDM across various study characteristics. High-quality studies confirmed the increased risk of preeclampsia with probiotic use. Increased GA was observed in studies of moderate quality, with lower doses and *Lactobacillus* and *Bifidobacterium* strains. Probiotics did not significantly impact cesarean section or gestational weight gain in both overall and subgroup analyses.

**Conclusion:**

Probiotics supplementation among pregnant women resulted in a notable reduction in the risk of GDM but an elevation in the risk of preeclampsia and an extension of GA.

## 1. Introduction

Pregnancy represents a crucial phase where complications can arise, with conditions such as gestational diabetes mellitus (GDM) and preeclampsia being significant concerns, impacting around 12% to 18% and 5% to 7% of pregnancies, respectively, with increasing prevalence globally in recent decades.^[1,2]^ Both GDM and preeclampsia have been associated with various adverse maternal and neonatal consequences, including maternal mortality, increased likelihood of requiring a cesarean section (CS), and changes in gestational age (GA).^[3,4]^ Pregnancy complications significantly impact maternal and child health, highlighting the urgent need for research and therapeutic interventions to mitigate health and socioeconomic consequences.

The etiology of pregnancy complications arises from an interactions among genetic, epigenetic, and environmental factors.^[1]^ Growing evidence also indicates that maternal gut microbiota dysbiosis can have causal effects on various pregnancy complications.^[5]^ During pregnancy, the maternal gut microbiome undergoes dynamic changes, marked by a substantial increase in pathogenic bacteria like *Actinobacteria* and *Proteobacteria*, alongside a decrease in beneficial bacterial species such as *Faecalibacterium prausnitzii* and *Roseburia intestinalis*.^[6]^ Metabolites derived from the gut microbiota play a role in numerous pathophysiological pathways, including gut microbiota-driven inflammation, metabolic dysregulation, and placental dysfunction, which are intricately linked to the development of pregnancy complications.^[7,8]^ Hence, the utilization of probiotics during pregnancy to regulate gut microbiota has surfaced as a promising strategy for the prevention of pregnancy complications. Probiotics are defined as live microorganisms that provide health advantages to the host when administered in sufficient amounts. Several randomized clinical trials (RCTs) have explored the impacts of probiotic supplementation during pregnancy on GDM, preeclampsia, CS, and GA, yielding conflicting outcomes.^[9,10]^ The results of recent meta-analyses of RCTs have been also inconsistent, and the overall impact of probiotics on pregnancy complications remains unclear. The study by Chu et el^[11]^ found that probiotics did not impact the risk of GDM but increased the risk of developing preeclampsia. In contrast, Pakmehr et al^[12]^ reported that probiotics act preventively against GDM, with no significant effect on preeclampsia risk. Variations in probiotic dose and strain, treatment duration, and sample sizes across previous studies may have contributed to the heterogeneity in results. This umbrella meta-analysis aimed to consolidate evidence from various systematic reviews and meta-analyses concerning the impact of maternal probiotic supplementation on pregnancy complications.

## 2. Materials and methods

This umbrella meta-analysis was conducted following the guidelines outlined in the PRISMA statement.^[13]^ Since this review used previously published data, ethical approval was not required for this type of study.

### 2.1. Search strategy

We conducted a systematic search for meta-analyses of pertinent clinical trial studies in PubMed and Scopus, covering the period from their inception to April 2024. The search strategy involved utilizing a combination as the following terms: (“Probiotics”[Majr] OR “Prebiotics”[Mesh] OR “Synbiotics” OR Probiotic*[Title/Abstract] OR Prebiotic*[Title/Abstract] OR synbiotic*[Title/Abstract] OR Lactobacillus[Title/Abstract] OR Bifidobacterium[Title/Abstract] OR Saccharomyces[Title/Abstract]) AND (pregnancy complications[Title/Abstract] OR gestational diabetes[Title/Abstract] OR GDM[Title/Abstract] OR preeclampsia[Title/Abstract] OR gestational age[Title/Abstract] OR Caesarean[Title/Abstract] OR “gestational weight gain”[Title/Abstract] OR “maternal weight gain”[Title/Abstract]) AND (meta-analysis[Title/Abstract]). The search was limited to English publications. We broadened our search scope by examining the citations of identified reviews. The publications were imported into EndNote software, and following the inclusion of manually searched results, 2 investigators separately evaluated the titles, abstracts, and full texts of the identified articles in 2 phases to select those meeting our eligibility criteria. To manage the obtained studies, the citations were imported into EndNote software. Two authors independently assessed the title/abstracts and full texts of the identified articles in 2 phases to select those meeting the eligibility criteria. Any discrepancies were addressed by a group discussion involving all authors.

### 2.2. Eligibility criteria

The criteria for inclusion were as outlined below: study type: meta-analyses of clinical trial studies; participants: the studied population was pregnant women; intervention: the intervention entailed the utilization of probiotics either on their own or in conjunction with prebiotics; comparator: any placebo/control; outcome: the primary outcomes of interest were risk of GDM and preeclampsia, while the secondary outcomes were CS, gestational weight gain (GWG), and GA at delivery. Review studies without a quantitative synthesis of data, animal studies, and studies with unrelated intervention/outcome were excluded. The evaluation of eligibility criteria was conducted by 2 independent investigators, and any disagreements were resolved through group discussion.

### 2.3. Data extraction

Two reviewers independently collected data using a data extraction sheet, and any disagreements were resolved through consensus. The data extracted comprised total number of included studies, duration of follow-ups, publication year, name of the 1st author, sample size, type and dose of intervention, outcomes, risk of bias (RoB), the percentage of RCTS with low RoB in each meta-analysis, and effect sizes. When data were not available in the original publications, we contacted the corresponding authors to obtain the data.

### 2.4. Methodological quality of studies

The quality of each meta-analysis was evaluated utilizing the “A Measurement Tool to Assess Systematic Reviews-2” criteria.^[14]^ This tool has 16 items and categorizes the quality of studies to high, moderate, low, and critically low.

### 2.5. Statistical analysis

We extracted summary effect sizes, specifically relative risks (RRs) for dichotomous outcomes and standardized mean differences (SMDs) for continuous outcomes, along with their corresponding 95% confidence intervals (CIs), from each of the 27 included meta-analyses. These summary estimates were subsequently pooled to perform a meta-analysis at the umbrella review level. *I*^2^ statistics was applied to measure heterogeneity among the studies and values above 50% suggested significant heterogeneity. The random-effects model was employed for analyses due to the predicted heterogeneity across the studies.^[15]^ Publication bias was evaluated with the use of Egger regression test. A *P*-value of <.1 showed statistically significant bias.^[16]^ In cases where publication bias was notable, a trim-and-fill method was employed to rectify the outcomes for the observed bias. To validate the results’ robustness, sensitivity analysis was done by removing 1 study at a time and analyzing other studies. To find the origins of the heterogeneity, subgroup analyses were conducted based on the quality of studies, sample size, duration of treatment, and dose and strain of probiotics. Meta-regression analyses were also conducted to assess the effect of sample size, follow-up duration, publication year, and the proportion of RCTs with low RoB in each meta-analysis on the polled estimates. The Stata version 13 (StataCorp, College Station), was used to carry out all analyses.

## 3. Results

### 3.1. Characteristics of studies

**Figure 1.**
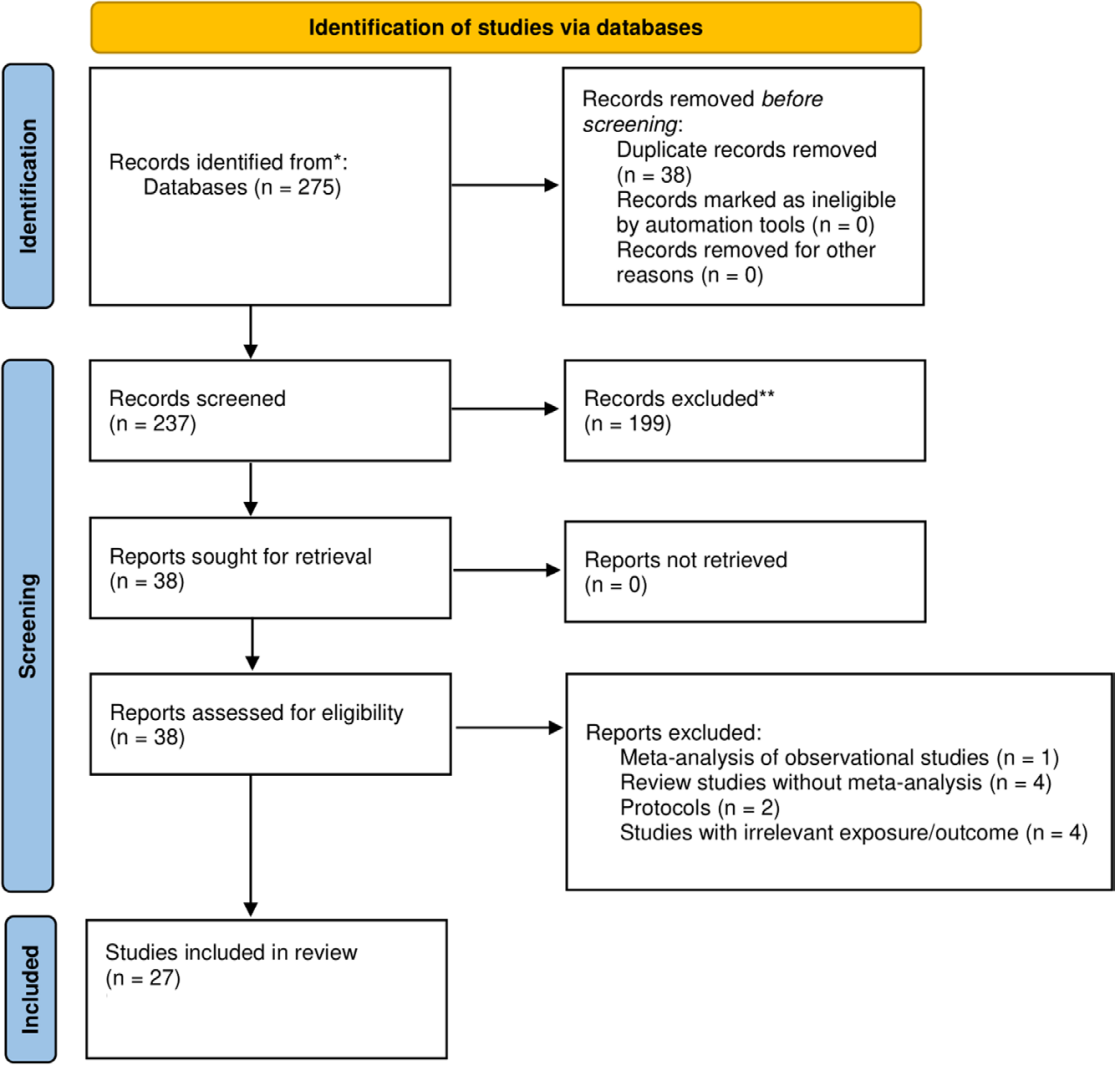
Flow diagram for the process of study selection

The systematic search found 275 publications. After removing 38 duplicates, we evaluated 237 titles/abstracts, leading to a total of 38 articles that underwent full-text evaluation. After full-text screening, 11 studies were excluded since they were meta-analysis of observational studies, review studies, protocols, and irrelevant publications in terms of supplementation/outcome. Finally, 27 studies,^[1,2,11,12,17–39]^ published during 2009 to 2024, were analyzed. Figure 1 presents the flow diagram of study selection. The sample size of the studies was between 402 and 16,545 subjects, with an overall 83,817 participants. The supplementation follow-ups among the publications ranged from 6 to 20 weeks. The dose of probiotics varied from 1.7 × 10^8^ to 16.8 × 10^10^ colony-forming units (CFU). The majority of studies assessed ROB of the analyzed RCTs using Cochrane tool,^[40]^ while 1 study applied Jadad score^[41]^ and 4 studies did not report RoB assessment. The proportion of studies with low ROB in meta-analyses varied from 10% to 100%. The intervention was mixed probiotics administered orally in all studies. However, some studies also presented the effect of specific strains of probiotics, including *Lactobacillus*, *Bifidobacterium*, *Streptococcus*, or their combinations on the outcomes. Data for GDM was reported in 15 studies,^[11,12,18,20,21,24,26,27,31–35,37,38]^ for preeclampsia in 10 studies,^[1,2,11,12,25,27,29,30,33,39]^ for CS in 10 studies,^[1,12,17,21,23,25,27–30]^ for GWG in 4 studies,^[21,25,27,36]^ and for GA in 10 studies.^[2,17,19,22,23,25,27,29,30,33]^ Other features of the analyzed papers are presented in Table 1.

**Table 1:** Characteristics of the included studies.

Study	Year	Country	Total included studies	Sample size	Supplementation	Risk of bias assessment, high quality/total studies	Percentage of primary studies with high quality	Follow-up	Dose of probiotics (CFU)	Outcomes	Quality
Zhang	2022	China	12	2213	Mixed probiotics	NR	NR	NR	NR	GDM	Low
Chu	2022	China	5	1048	Mixed probiotics	NR	NR	NR	NR	GDM, Preeclampsia	Moderate
Chen	2023	China	6	2284	Mixed probiotics	NR	NR	NR	NR	GDM, Preeclampsia, gestational age at delivery	Low
Zhang	2019	China	11	719	Mixed probiotics	Cochrane tool, 10/11	90	6 wk	6.47 × 10^9^	Gestational age at delivery	High
Yefet	2023	Israel	14	854	Mixed probiotics, *Lactobacillus*, *Bifidobacterium*	Cochrane tool, 6/14	42	7 wk	1.97 × 10^10^	Gestational weight gain	Moderate
Takele	2024	Australia	5	1104	Mixed probiotics	Cochrane tool, 2/5	40	NR	NR	GDM	High
Tabatabaeizadeh	2022	Iran	4	533	Mixed probiotics	Cochrane tool, 3/4	75	NR	1.7 × 10^8^	GDM	Low
Wu	2024	China	15	1006	Mixed probiotics, *Lactobacillus*, *Lactobacillus* + *Bifidobacterium*, *Lactobacillus* + *Bifidobacterium* + *Streptococcus*	Cochrane tool, 13/15	86	6 wk	1.8 × 10^10^	Preeclampsia, cesarean section	Moderate
McDougall	2024	Australia	29	7735	Mixed probiotics	Cochrane tool, 5/29	17	NR	NA	Preeclampsia, gestational age at delivery	Moderate
Pakmehr	2022	Iran	10	2921	Mixed probiotics, single strain, multiple strain	Cochrane tool, 1/10	10	20 wk	16.8 × 10^10^	GDM, preeclampsia, cesarean section	High
Movaghar	2022	Iran	5	402	Mixed probiotics	Cochrane tool, 5/5	100	7 wk	2.42 × 10^10^	Preeclampsia, cesarean section, gestational age at delivery	Moderate
Masulli	2020	Italy	17	5123	Mixed probiotics	Cochrane tool, 17/17	100	13 wk	2.33 × 10^10^	GDM	Moderate
Han	2018	China	10	1139	Mixed probiotics	Cochrane tool, 10/10	100	8 wk	4.22 × 10^9^	GDM, cesarean section, gestational weight gain	High
Dugoua	2009	Canada	8	1546	*Lactobacillus* + *Bifidobacterium* + *Streptococcus*	NR	NR	NR	1.24 × 10^10^	Cesarean section, gestational age at delivery	Low
Chan	2021	China	53	9443	Mixed probiotics	Cochrane tool, NR	NR	7 wk	13.6 × 10^10^	GDM	Moderate
Kuang	2020	China	18	4356	Mixed probiotics, *Lactobacillus*	Jadad score, 17/18	94	14 wk	1.21 × 10^10^	Cesarean section, gestational age at delivery	Moderate
Li	2024	China	14	3527	Mixed probiotics	Cochrane tool, 12/14	85	19 wk	1.43 × 10^10^	GDM	Moderate
Lim	2023	Australia	5	1104	Mixed probiotics	Cochrane tool, 1/5	20	15 wk	NR	GDM	High
Pérez-Castillo	2021	Spain	46	8519	Mixed probiotics	Cochrane tool, 11/46	23	7 wk	1.28 × 10^10^	Cesarean section	Moderate
Hao-zhao	2021	China	12	894	Mixed probiotics	Cochrane tool, NR	NR	7 wk	2.24 × 10^10^	Preeclampsia, cesarean section, gestational age at delivery	Moderate
Valiati	2023	Brazil	6	593	Mixed probiotics	Cochrane tool, 3/6	50	12 wk	9.45 × 10^10^	Preeclampsia	High
Tang	2022	China	46	16,545	Mixed probiotics	Cochrane tool, 46/46	100	6 wk	NR	GDM	High
Rogozińska	2015	United Kingdom	20	6444	Mixed probiotics	Cochrane tool, 8/20	40	NR	NR	GDM	Moderate
Chatzakis	2019	Greece	23	4237	Mixed probiotics	Cochrane tool, 21/23	91	17 wk	NA	GDM	High
Grev	2018	USA	4	518	Mixed probiotics, *Lactobacillus*	Cochrane tool, 3/4	75	6 wk	13.1 × 10^10^	Gestational age at delivery	High
Okesene-Gafa	2020	New Zealand	9	695	Mixed probiotics	Cochrane tool, 9/9	100	7 wk	14 × 10^10^	Preeclampsia, cesarean section, gestational weight gain, gestational age at delivery	High
Davidson	2021	Australia	7	1647	Mixed probiotics, *Lactobacillus*, *Lactobacillus* + *Bifidobacterium*	Cochrane tool, 7/7	100	18 wk	1.6 × 10^10^	GDM, preeclampsia, cesarean section, gestational weight gain, gestational age at delivery	High

CFU = colony-forming unit, GDM = gestational diabetes mellitus, NR = not reported.

### 3.2. Quality of the studies

Comprehensive details regarding the methodological qualities of the studies, assessed according to the A Measurement Tool to Assess Systematic Reviews-2 criteria, are reported in Table S1, Supplemental Digital Content, https://links.lww.com/MD/Q895. The quality of studies was rated as high, moderate, and low in 11, 12, and 4 studies, respectively (Table S1, Supplemental Digital Content, https://links.lww.com/MD/Q895).

### 3.3. Results of the umbrella meta-analysis

**Figure 2.**
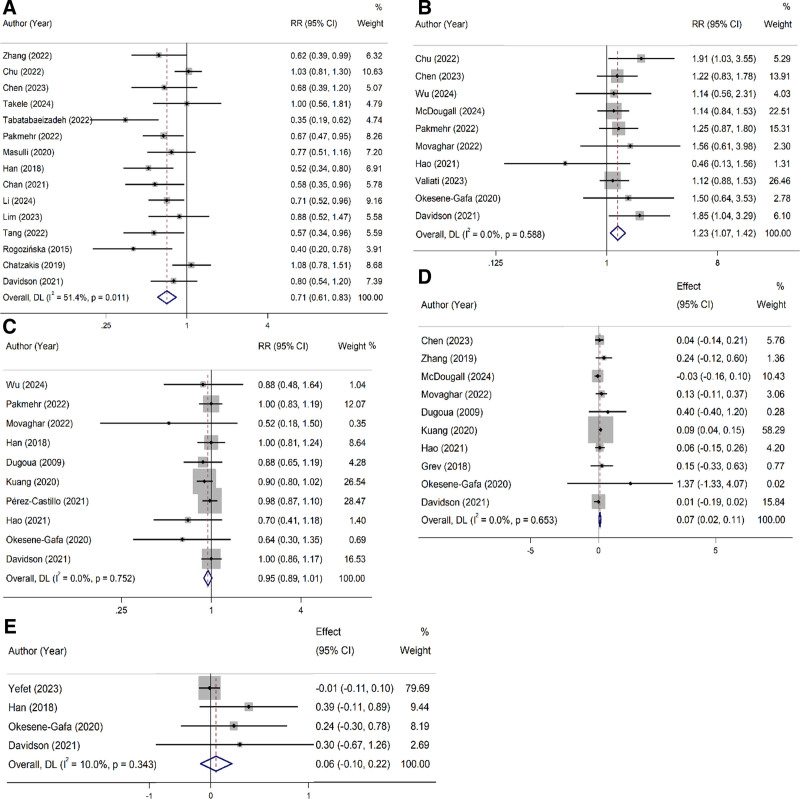
Pooled analysis for the effect of probiotics during pregnancy on gestational diabetes (A), preeclampsia (B), cesarean section (C), gestational age (D), and gestational weight gain (E).

In the overall analysis, probiotics supplementation was linked to a significantly reduction in GDM risk (RR = 0.71, 95% CI: 0.61–0.83; *P*_heterogeneity_: .01), but a higher risk of preeclampsia (RR = 1.23, 95% CI: 1.07–1.42; *P*_heterogeneity_: .58) and an increase in GA (SMD = 0.07, 95% CI: 0.02–0.11; *P*_heterogeneity_: .65) (Fig. 2). In the subgroup analysis, the preventive effect of probiotics on GDM was supported by all subgroups based on the quality of studies, sample size, and dose and duration of supplementation. The increased risk of preeclampsia following the probiotics use was confirmed by high-quality studies (RR = 1.25, 95% CI: 1.02–1.53). For GA, an increased GA was observed in studies of moderate quality (SMD = 0.07, 95% CI: 0.02–0.12), probiotics doses of < 2 × 10^10^ CFU (SMD = 0.08, 95% CI: 0.02–0.13), and when either *Lactobacillus* (SMD = 0.10, 95% CI: 0.03–0.16) or *Bifidobacterium* (SMD = 0.30, 95% CI: 0.03–0.57) was administered. Probiotics did not show a significant effect on CS and GWG in both the overall and subgroup analyses (Table 2).

**Table 2:** Overall and subgroup analyses for the effect of probiotics during pregnancy on pregnancy outcomes.

				Test of effect	Test of heterogeneity	
Categorical Outcomes		Subgroups	Studies	RR (95%CI)	*I*^2^ (%)	*P*-value
Gestational diabetes		Overall	15	0.71 (0.61 to 0.83)	51.4	0.01
	Quality	Low	3	0.54 (0.37 to 0.79)	34.3	0.21
		Moderate	5	0.72 (0.55 to 0.96)	62.0	0.03
		High	7	0.77 (0.62 to 0.95)	41.2	0.11
	Sample size	≥2000 participants	9	0.70 (0.58 to 0.84)	35.2	0.14
		<2000 participants	6	0.67 (0.47 to 0.94)	58.3	0.04
	Dose of supplementation	NR	8	0.80 (0.63 to 1.00)	50.5	0.04
		≥2 × 10^10^ CFU	3	0.68 (0.54 to 0.86)	0.0	0.69
		<2 × 10^10^ CFU	4	0.60 (0.44 to 0.82)	54.0	0.08
	Follow-up duration	NR	6	0.65 (0.45 to 0.95)	72.0	0.003
		≥8 wk	7	0.76 (0.64 to 0.91)	28.7	0.21
		<8 wk	2	0.58 (0.40 to 0.83)	0.0	0.96
	Type of probiotics	*Lactobacillus*	2	0.84 (0.40 to 1.75)	59.2	0.12
		*Bifidobacterium*	1	1.26 (0.56 to 2.83)	–	–
		*Lactobacillus* plus *Bifidobacterium*	1	0.83 (0.50 to 1.37)	–	–
Preeclampsia		Overall	10	1.23 (1.07 to 1.42)	0.0	0.58
	Quality	Low	1	1.22 (0.83 to 1.79)	–	–
		Moderate	5	1.23 (0.91 to 1.67)	18.0	0.30
		High	4	1.25 (1.02 to 1.53)	0.0	0.46
	Sample size	≥2000 participants	3	1.18 (0.94 to 1.49)	0.0	0.70
		<2000 participants	7	1.23 (0.97 to 1.55)	5.1	0.38
	Dose of supplementation	NR	3	1.25 (0.99 to 1.58)	8.3	0.33
		≥2 × 10^10^ CFU	5	1.17 (0.95 to 1.44)	0.0	0.58
		<2 × 10^10^ CFU	2	1.52 (0.95 to 2.42)	7.4	0.29
	Follow-up duration	NR	3	1.25 (0.99 to 1.58)	8.3	0.33
		≥8 wk	3	1.25 (0.99 to 1.58)	8.3	0.33
		<8 wk	4	1.17 (0.75 to 1.82)	0.0	0.41
Caesarian section		Overall	10	0.95 (0.89 to 1.01)	0.0	0.75
	Quality	Low	1	0.88 (0.65 to 1.19)	-	-
		Moderate	5	0.93 (0.86 to 1.01)	0.0	0.50
		High	4	0.99 (0.90 to 1.10)	0.0	0.72
	Sample size	≥2000 participants	3	0.95 (0.88 to 1.03)	0.0	0.51
		<2000 participants	7	0.95 (0.86 to 1.06)	0.0	0.60
	Dose of supplementation	≥2 × 10^10^ CFU	4	0.86 (0.66 to 1.11)	21.8	0.28
		<2 × 10^10^ CFU	6	0.95 (0.89 to 1.02)	0.0	0.85
	Follow-up duration	NR	1	0.88 (0.65 to 1.19)	-	-
		≥8 wk	4	0.96 (0.88 to 1.04)	0.0	0.64
		<8 wk	5	0.95 (0.85 to 1.06)	0.0	0.41
	Type of probiotics	*Lactobacillus*	3	1.04 (0.88 to 1.23)	0.0	0.89
		*Lactobacillus* plus *Bifidobacterium*	2	0.84 (0.56 to 1.25)	63.3	0.09
		*Lactobacillus* plus *Bifidobacterium* and *Streptococcus*	2	1.30 (0.53 to 3.17)	78.3	0.03

Continuous outcomes		Subgroups	Studies	SMD (95%CI)	*I*^2^ (%)	*P*
Gestational weight gain		Overall	4	0.06 (-0.10 to 0.22)	10.0	0.34
	Quality	Moderate	1	-0.01 (-0.11 to 0.10)	–	–
		High	3	0.32 (-0.02 to 0.66)	0.0	0.92
	Sample size	<2000 participants	4	0.06 (-0.10 to 0.22)	10.0	0.34
	Dose of supplementation	≥2 × 10^10^ CFU	1	0.24 (-0.30 to 0.78)	–	–
		<2 × 10^10^ CFU	3	0.09 (-0.16 to 0.33)	25.3	0.26
	Follow-up duration	≥8 wk	2	0.37 (-0.07 to 0.71)	0.0	0.87
		<8 wk	2	0.00 (-0.10 to 0.10)	0.0	0.37
Gestational age at delivery		Overall	10	0.07 (0.02 to 0.11)	0.0	0.65
	Quality	Low	2	0.08 (-0.11 to 0.23)	0.0	0.39
		Moderate	4	0.07 (0.02 to 0.12)	3.0	0.76
		High	4	0.04 (-0.08 to 0.13)	0.0	0.64
	Sample size	≥2000 participants	3	0.05 (-0.07 to 0.16)	64.0	0.09
		<2000 participants	7	0.05 (-0.03 to 0.14)	0.0	0.69
	Dose of supplementation	NR	2	-0.01 (-0.11 to 0.10)	0.0	0.52
		≥2 × 10^10^ CFU	4	0.10 (-0.05 to 0.25)	0.0	0.77
		<2 × 10^10^ CFU	4	0.08 (0.02 to 0.13)	5.8	0.39
	Follow-up duration	NR	3	0.00 (-0.10 to 0.11)	0.0	0.50
		≥8 wk	2	0.08 (-0.01 to 0.14)	42.4	0.18
		<8 wk	5	0.12 (-0.02 to 0.28)	0.0	0.80
	Type of probiotics	*Lactobacillus*	2	0.10 (0.03 to 0.16)	0.0	0.73
		*Bifidobacterium*	1	0.30 (0.03 to 0.57)	–	–
		*Lactobacillus* plus *Bifidobacterium* and *Streptococcus*	1	0.40 (-0.40 to 1.20)	–	–
		*Lactobacillus*	1	0.07 (-0.09 to 0.23)	–	–

### 3.4. Sensitivity analysis

In the sensitivity analysis, the pooled effect sizes for GDM, preeclampsia, CS, and GWG were not affected by individual studies, however, the pooled estimate for GA was significantly affected (SMD = 0.03, 95% CI: −0.03 to 0.09) when the study by Kuang et al^[23]^ was removed from the main analysis.

### 3.5. Meta-regression analysis

In the meta-regression analysis, the results were not influenced by the proportion of RCTs with low RoB in each meta-analysis, publication year, sample size, and follow-up duration.

### 3.6. Publication bias

**Figure 3.**
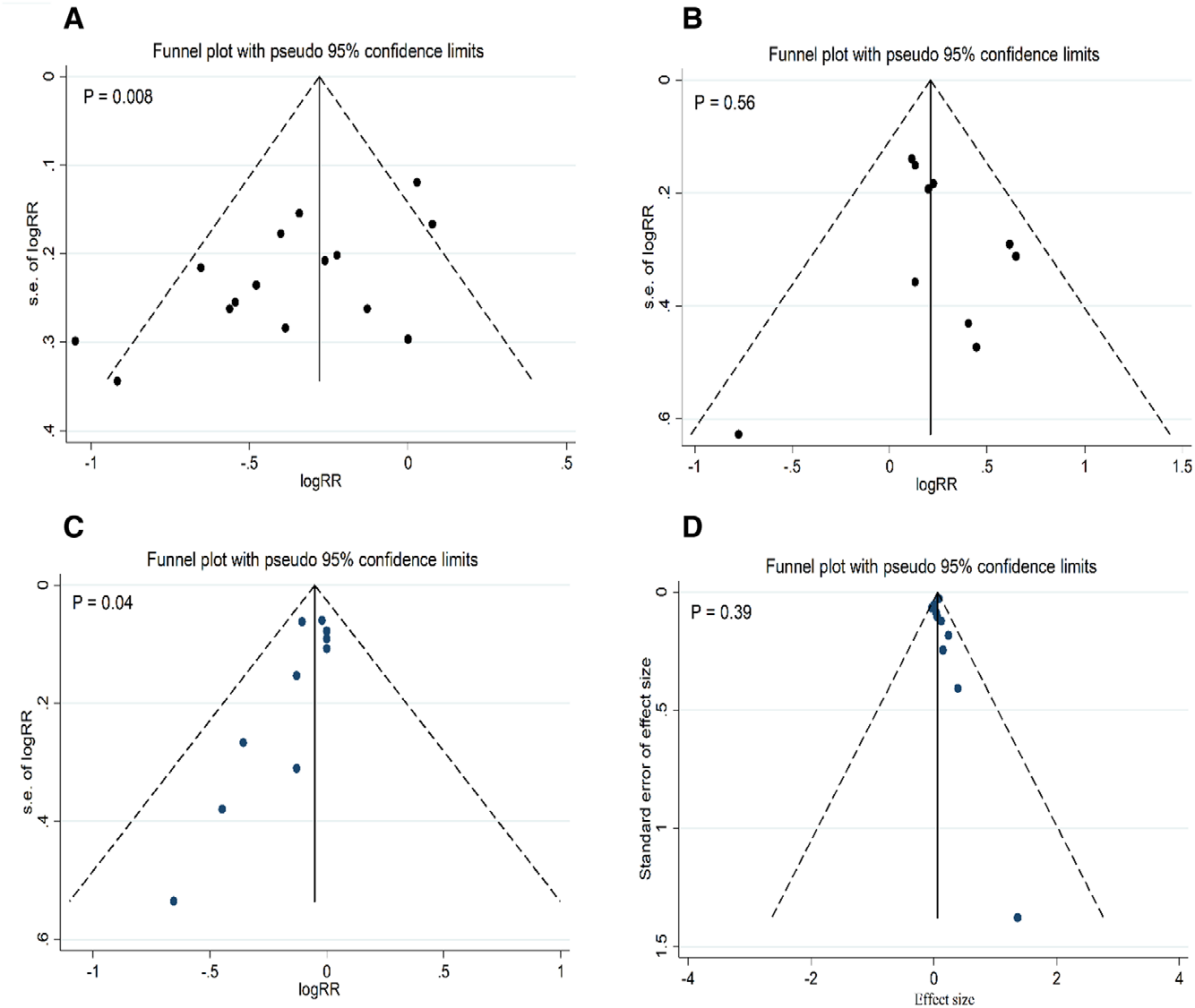
Funnel plots for publication bias in studies examining the effect of probiotics during pregnancy on gestational diabetes (A), preeclampsia (B), cesarean section (C), and gestational age (D).

There was no publication bias for preeclampsia and GA. A significant publication bias was detected for GDM and CS (Fig. 3). The trim-and-fill adjustment for publication bias did not significantly change the pooled effect sizes for GDM and CS, suggesting the reliability of the findings.

## 4. Discussion

This umbrella meta-analysis investigated the influence of probiotics consumption on pregnancy complications. The analysis revealed that administering probiotics to pregnant women effectively reduces the risk of GDM but notably raises the risk of preeclampsia. Moreover, probiotics were associated with an increase in GA. No significant effect was observed for CS and GWG.

The effect of probiotics on pregnancy complications has been inconclusive.^[11,12]^ The heterogeneity in probiotic strains, and dosages and duration of intervention could explain the contradictory results. At present, there is inadequate evidence to support recommending routine supplementation for pregnant women. In guidelines of the World Health Organization, the International Federation of Gynecology and Obstetrics, and the USA Academy of Nutrition and Dietetics, probiotics are not noted to be used during pregnancy.^[2]^ The Australian Pregnancy Care Guidelines also acknowledged that while probiotics may have favorable impacts, they are not recommended because of the unclear advantages of routine supplementation in pregnancy.^[2,42]^ Our analysis revealed that probiotics could reduce the risk of GDM while increase the risk of preeclampsia and GA in pregnant women. In agreement with our findings, a recent Cochrane review revealed high-certainty evidence indicating that probiotics used for preventing GDM significantly raised the risk of preeclampsia in pregnant women.^[27]^ This suggests a need for further investigation and caution when considering probiotic interventions in pregnancy. Based on the current evidence, the impact on GA appears to be minimal, with 0.07 week increase in GA following the treatment with probiotics. While statistically significant, this magnitude is very small and likely not clinically meaningful in most cases. Such a minimal increase in gestational length is unlikely to have a substantial impact on neonatal or maternal outcomes. However, it is important to recognize this finding and interpret it with caution in the clinical context. The mechanisms underlying the increased risk of preeclampsia and GA in pregnant women receiving probiotics remain unclear. While some studies suggest potential beneficial effects of probiotics on systemic inflammation and placental function, the biological pathways leading to an increased risk have not been well elucidated. More high-quality mechanistic studies and clinical trials are needed to fully understand these potential adverse effects and clarify the mechanisms by which probiotics may influence such pregnancy outcomes. Probiotic supplementation was associated with a significant 29% reduction in the risk of GDM, which is clinically meaningful given the well-established risks of GDM for both maternal and offspring health, including increased risk of type 2 diabetes and adverse perinatal outcomes. However, probiotics were also linked to a 23% increased risk of preeclampsia, a serious pregnancy complication associated with significant maternal and fetal morbidity. These opposing effects highlight the need for cautious interpretation and further research to optimize probiotic use during pregnancy for maximal benefit and minimal harm.

The positive preventive effects of probiotics on GDM can be mediated by several mechanisms, including modulation of gut microbiota, increased production of short-chain fatty acids, regulation of inflammatory markers, improved insulin sensitivity, enhanced glucagon-like peptide-1 (GLP-1) production, reduced lipopolysaccharide (LPS) levels, increased production of peptide YY, and enhanced regulatory T cell (Treg) activity.^[43–45]^ Probiotics alter the composition of the gut microbiota, leading to changes in the metabolic pathways and the regulation of glucose metabolism. This modulation can reduce the risk of GDM by improving insulin sensitivity and glucose tolerance.^[43]^ Probiotics stimulate the production of short-chain fatty acids, which can improve insulin sensitivity and reduce inflammation, thereby reducing the risk of GDM.^[45]^ Probiotics have been shown to reduce blood glucose levels in both animal and human studies, which can help prevent or manage GDM.^[46]^ Evidence has shown that the expression of Glut-4, an insulin-regulated glucose transporter, is increased after probiotics supplementation, which enhance insulin signaling pathways.^[47]^ Probiotics have anti-inflammatory effects, reducing the levels of inflammatory markers such as tumor necrosis factor-alpha, high-sensitivity C-reactive protein, and interleukin 6.^[44]^ This reduction in inflammation can help in maintaining glucose homeostasis and reducing the risk of GDM. Studies have suggested that probiotics stimulate the production of GLP-1, a hormone that plays a crucial role in glucose regulation.^[48]^ GLP-1 has been shown to be negatively associated with GDM.^[49]^ The levels of LPS in the gut is reduced following the probiotics administration; LPS can contribute to the development of insulin resistance and GDM. Lower LPS levels could improve glucose and insulin homeostasis.^[45]^ Probiotics can also stimulate the production of peptide YY, a hormone that helps in glucose regulation.^[36]^ Probiotics enhance Treg activity, which helps in maintaining immune homeostasis and reducing inflammation. Enhanced Treg activity decrease the risk of GDM by maintaining glucose homeostasis.^[50]^ These biological mechanisms collectively contribute to the positive preventive effects of probiotics on GDM, making them a promising intervention for reducing the risk of this condition.

This is the 1st umbrella meta-analysis examining the effect of probiotics on pregnancy complications. The study’s strengths encompassed unrestricted searches across time and languages, a large number of analyzed studies with relatively high sample sizes, and thorough subgroup analyses and meta-regression to identify potential influencing factors and sources of heterogeneity. Furthermore, the Grading of Recommendations, Assessment, Development, and Evaluation system was applied to grade the strength of evidence and the strength of recommendations. Several limitations of this study need to be taken into account. First, a noteworthy heterogenetic was observed among the studies on GDM, which may limit the generalizability of the findings. In the subgroup analysis, variability in study quality, sample size, and dose and duration of interventions were found as the sources of the heterogeneity. This heterogeneity could result from the variability in probiotic dosages, ranging from 1.7 × 10^8^ to 16.8 × 10^10^ CFU, and differences in formulations, including single-strain, multi-strain, and combinations with or without prebiotics. This variability may affect the generalizability of the findings and could not be fully controlled for in the analyses. Although subgroup analyses were conducted to explore potential sources of the high heterogeneity observed for GDM as the primary outcome, the overall estimate should be interpreted with caution. Second, despite the absence of time or language restrictions in the search, there was remarkable publication bias for GDM and CS, showing that some smaller studies with null or negative results might be missing, potentially skewing the overall effect size. However, the trim-and-fill analysis suggesting the findings were robust is reassuring. Third, the studies included in this analysis examined the use of mixed probiotics on the outcomes, and the findings of the subgroup analysis for probiotic strains should be approached with caution due to the limited number of studies analyzed. Moreover, the effect of probiotics on pregnancy outcomes could vary across different trimesters. The timing of intervention was not adequately explored in the included studies. This leads to lack of strain-specific and timing-specific conclusions. Further studies are required to assess the strain-specific effects of probiotics and the influence of intervention timing on pregnancy complications.

In conclusion, this umbrella meta-analysis indicated that probiotic intervention may be a promising preventive approach for GDM; however the optimal strain and initiation timing of probiotics administration are yet to be established. Nevertheless, pregnant women receiving probiotics are at increased risk of preeclampsia and elevated GA, but subgroup analyses show that the effect on GA was specific to lower doses (<2 × 10^10^ CFU). This suggests that while probiotics may help prevent GDM, they may also have other potential complications that need to be considered and monitored closely during pregnancy. The effects of probiotics on pregnancy outcomes are likely influenced by probiotic dose, duration of supplementation, strain specificity, and timing of administration during pregnancy. However, the limited number of trials, high heterogeneity among studies, and varying probiotic regimens highlight the need for further well-designed trials to clarify these dose-, duration-, strain-, and timing-specific effects. Further studies are also needed to confirm the negative effect of probiotics on preeclampsia and GA and to better understand the mechanisms by which probiotics affect these outcomes.

## Author contributions

**Conceptualization:** Fengling Li.

**Data curation:** Qian Gao, Yuanju Sun, Yuanyuan Qu.

**Investigation:** Qian Gao, Yuanju Sun.

**Methodology:** Qian Gao, Yuanyuan Qu, Pan Li.

**Resources:** Yuanyuan Qu, Fengling Li.

**Software:** Qian Gao, Yuanyuan Qu, Pan Li.

**Supervision:** Pan Li.

**Writing – original draft:** Qian Gao, Yuanju Sun, Yuanyuan Qu.

**Writing – review & editing:** Fengling Li, Pan Li.

## Supplementary Material



## References

[1] Wu R Luan J Hu J Li Z. Effect of probiotics on pregnancy outcomes in gestational diabetes: systematic review and meta-analysis. Arch Gynecol Obstet. 2024;310:769–81. 38236281 10.1007/s00404-023-07346-5

[2] McDougall A Nguyen R Nguyen P-Y. The effects of probiotics administration during pregnancy on preeclampsia and associated maternal, fetal, and newborn outcomes: a systematic review and meta-analysis. Am J Obstetrics Gynecol MFM. 2024;6:101322. 10.1016/j.ajogmf.2024.101322 38447676

[3] Johns EC Denison FC Norman JE Reynolds RM. Gestational diabetes mellitus: mechanisms, treatment, and complications. Trends Endocrinol Metab. 2018;29:743–54. 30297319 10.1016/j.tem.2018.09.004

[4] Minire A Mirton M Imri V Lauren M Aferdita M. Maternal complications of preeclampsia. Med Arch. 2013;67:339–41. 24601166 10.5455/medarh.2013.67.339-341

[5] Li C Liu C Li N. Causal associations between gut microbiota and adverse pregnancy outcomes: a two-sample Mendelian randomization study. Front Microbiol. 2022;13:1059281. 36590417 10.3389/fmicb.2022.1059281 PMC9801412

[6] Koren O Goodrich JK Cullender TC. Host remodeling of the gut microbiome and metabolic changes during pregnancy. Cell. 2012;150:470–80. 22863002 10.1016/j.cell.2012.07.008 PMC3505857

[7] Taylor BL Woodfall GE Sheedy KE. Effect of probiotics on metabolic outcomes in pregnant women with gestational diabetes: a systematic review and meta-analysis of randomized controlled trials. Nutrients. 2017;9:461. 28475161 10.3390/nu9050461 PMC5452191

[8] Di Simone N Santamaria Ortiz A Specchia M. Recent insights on the maternal microbiota: impact on pregnancy outcomes. Front Immunol. 2020;11:528202. 33193302 10.3389/fimmu.2020.528202 PMC7645041

[9] Callaway LK McIntyre HD Barrett HL. Probiotics for the prevention of gestational diabetes mellitus in overweight and obese women: findings from the SPRING double-blind randomized controlled trial. Diabetes Care. 2019;42:364–71. 30659070 10.2337/dc18-2248

[10] Nitert MD Barrett HL Foxcroft K. SPRING: an RCT study of probiotics in the prevention of gestational diabetes mellitus in overweight and obese women. BMC Pregnancy Childbirth. 2013;13:1–7. 23442391 10.1186/1471-2393-13-50 PMC3585705

[11] Chu X Yan P Zhang N. Probiotics for preventing gestational diabetes mellitus in overweight or obese pregnant women: a systematic review and meta-analysis. Clin Nutr ESPEN. 2022;50:84–92. 35871956 10.1016/j.clnesp.2022.05.007

[12] Pakmehr A Ejtahed H-S Shirzad N Hemmatabadi M Farhat S Larijani B. Preventive effect of probiotics supplementation on occurrence of gestational diabetes mellitus: a systematic review and meta-analysis of randomized controlled trials. Front Med. 2022;9:1031915. 10.3389/fmed.2022.1031915 PMC9751955 36530883

[13] Parums DV. Review articles, systematic reviews, meta-analysis, and the updated preferred reporting items for systematic reviews and meta-analyses (PRISMA) 2020 guidelines. Med Sci Monit. 2021;27:e934475–1. 34421116 10.12659/MSM.934475 PMC8394590

[14] Lu C Lu T Ge L Yang N Yan P Yang K. Use of AMSTAR-2 in the methodological assessment of systematic reviews: protocol for a methodological study. Annal Transl Med. 2020;8:652–652. 10.21037/atm-20-392a PMC7290613 32566589

[15] DerSimonian R Kacker R. Random-effects model for meta-analysis of clinical trials: an update. Contemp Clin Trials. 2007;28:105–14. 16807131 10.1016/j.cct.2006.04.004

[16] Lin L Chu H. Quantifying publication bias in meta-analysis. Biometrics. 2018;74:785–94. 29141096 10.1111/biom.12817 PMC5953768

[17] Dugoua JJ Machado M Zhu X Chen X Koren G Einarson TR. Probiotic safety in pregnancy: a systematic review and meta-analysis of randomized controlled trials of *Lactobacillus*, *Bifidobacterium*, and *Saccharomyces* spp. J Obstetrics Gynaecol Canada: JOGC = Journal d'obstetrique et gynecologie du Canada: JOGC. 2009;31:542–52. 10.1016/S1701-2163(16)34218-9 19646321

[18] Rogozińska E Chamillard M Hitman GA Khan KS Thangaratinam S. Nutritional manipulation for the primary prevention of gestational diabetes mellitus: a meta-analysis of randomised studies. PLoS One. 2015;10:e0115526. 25719363 10.1371/journal.pone.0115526 PMC4342242

[19] Grev J Berg M Soll R. Maternal probiotic supplementation for prevention of morbidity and mortality in preterm infants. Cochrane Database Syst Rev. 2018;12:CD012519. 30548483 10.1002/14651858.CD012519.pub2 PMC6516999

[20] Chatzakis C Goulis DG Mareti E. Prevention of gestational diabetes mellitus in overweight or obese pregnant women: a network meta-analysis. Diabetes Res Clin Pract. 2019;158:107924. 31738997 10.1016/j.diabres.2019.107924

[21] Han MM Sun JF Su XH. Probiotics improve glucose and lipid metabolism in pregnant women: a meta-analysis. Annal Transl Med. 2019;7:99. 10.21037/atm.2019.01.61 PMC6462661 31019949

[22] Zhang J Ma S Wu S Guo C Long S Tan H. Effects of probiotic supplement in pregnant women with gestational diabetes mellitus: a systematic review and meta-analysis of randomized controlled trials. J Diabetes Res. 2019;2019:5364730. 31583250 10.1155/2019/5364730 PMC6748202

[23] Kuang L Jiang Y. Effect of probiotic supplementation in pregnant women: a meta-analysis of randomised controlled trials. Br J Nutr. 2020;123:870–80. 31856928 10.1017/S0007114519003374

[24] Masulli M Vitacolonna E Fraticelli F Della Pepa G Mannucci E Monami M. Effects of probiotic supplementation during pregnancy on metabolic outcomes: a systematic review and meta-analysis of randomized controlled trials. Diabetes Res Clin Pract. 2020;162:108111. 32194215 10.1016/j.diabres.2020.108111

[25] Okesene-Gafa KAM Moore AE Jordan V McCowan L Crowther CA. Probiotic treatment for women with gestational diabetes to improve maternal and infant health and well-being. Review. Cochrane Database Syst Rev. 2020;2020:Cd012970. 10.1002/14651858.CD012970.pub2 PMC7386668 32575163

[26] Chan KY Wong MMH Pang SSH Lo KKH. Dietary supplementation for gestational diabetes prevention and management: a meta-analysis of randomized controlled trials. Arch Gynecol Obstet. 2021;303:1381–91. 33745021 10.1007/s00404-021-06023-9

[27] Davidson SJ Barrett HL Price SA Callaway LK Nitert MD. Probiotics for preventing gestational diabetes. Cochrane Database Syst Rev. 2021;4:CD009951. 33870484 10.1002/14651858.CD009951.pub3 PMC8094741

[28] Pérez-Castillo M Fernández-Castillo R Lasserrot-Cuadrado A Gallo-Vallejo JL Rojas-Carvajal AM Aguilar-Cordero MJ. Reporting of perinatal outcomes in probiotic randomized controlled trials. a systematic review and meta-analysis. Nutrients. 2021;13:256. 33477352 10.3390/nu13010256 PMC7830438

[29] Zhou L Ding C Wu J. Probiotics and synbiotics show clinical efficacy in treating gestational diabetes mellitus: a meta-analysis. Primary Care Diabetes. 2021;15:937–47. 34417122 10.1016/j.pcd.2021.08.005

[30] Movaghar R Farshbaf-Khalili A Hajizade K MirzaRezaei ME Shahnazi M. The effect of probiotics or synbiotics on the hypertensive disorders of pregnant women with gestational diabetes: a systematic review and meta-analysis. J Caring Sci. 2022;11:94–104. 35919277 10.34172/jcs.2021.027 PMC9339131

[31] Tang Q Zhong Y Xu C Li W Wang H Hou Y. Effectiveness of five interventions used for prevention of gestational diabetes: a network meta-analysis. Medicine (Baltimore). 2022;101:e29126. 35475799 10.1097/MD.0000000000029126 PMC9276162

[32] Zhang L Hu X Wang Y. Effects of probiotic supplementation on glucose metabolism in pregnant women without diabetes: a systematic review and meta-analysis. Food Function. 2022;13:8388–98. 35856090 10.1039/d1fo04333a

[33] Chen X Pan L Zhang Z Niu R Zhang H Ma T. Probiotic supplement for the prevention of gestational diabetes: a meta-analysis of randomized controlled trials. Review. Z Geburtshilfe Neonatol. 2023;227:24–30. 36368685 10.1055/a-1956-3927

[34] Lim S Takele WW Vesco KK Redman L Josefson J. A systematic review and meta-analysis of participant characteristics in the prevention of gestational diabetes: a summary of evidence for precision medicine. medRxiv [Preprint]. 2023. doi: 10.1101/2023.04.16.23288650. 10.1038/s43856-023-00366-x PMC10551015 37794119

[35] Tabatabaeizadeh SA Tafazoli N. Effect of probiotic yogurt on gestational diabetes mellitus: a systematic review and meta-analysis. Diabetes Metabol Syndrome. 2023;17:102758. 10.1016/j.dsx.2023.102758 37062185

[36] Yefet E Bar L Izhaki I. Effects of probiotics on glycemic control and metabolic parameters in gestational diabetes mellitus: systematic review and meta-analysis. Nutrients. 2023;15:1633. 37049473 10.3390/nu15071633 PMC10097303

[37] Li X Zhang L He Y Zhang D Zhang S. Probiotics for the prevention of gestational diabetes mellitus: a meta-analysis of randomized controlled trials. Biomol Biomed. 2024;24:1092–104. 38642385 10.17305/bb.2024.10377 PMC11378997

[38] Takele WW Vesco KK Josefson J. Effective interventions in preventing gestational diabetes mellitus: a systematic review and meta-analysis. Commun Med. 2024;4:75. 38643248 10.1038/s43856-024-00491-1 PMC11032369

[39] Valiati N Puel EM Stefani CM Lataro RM. Does probiotic ingestion reduce the risk of preeclampsia? A systematic review. Appl Physiol Nutr Metabol = Physiologie appliquee, nutrition et metabolisme. 2024;49:135–47. 10.1139/apnm-2023-0089 37844331

[40] Jørgensen L Paludan-Müller AS Laursen DR. Evaluation of the Cochrane tool for assessing risk of bias in randomized clinical trials: overview of published comments and analysis of user practice in Cochrane and non-Cochrane reviews. Syst Rev. 2016;5:1–13. 27160280 10.1186/s13643-016-0259-8 PMC4862216

[41] Clark HD Wells GA Huët C. Assessing the quality of randomized trials: reliability of the Jadad scale. Control Clin Trials. 1999;20:448–52. 10503804 10.1016/s0197-2456(99)00026-4

[42] Tsakiridis I Kasapidou E Dagklis T. Nutrition in pregnancy: a comparative review of major guidelines. Obstetrical Gynecol Survey. 2020;75:692–702. 10.1097/OGX.0000000000000836 33252699

[43] Zheng Q-X Jiang X-M Wang H-W. Probiotic supplements alleviate gestational diabetes mellitus by restoring the diversity of gut microbiota: a study based on 16S rRNA sequencing. J Microbiol (Seoul, Korea). 2021;59:827–39. 10.1007/s12275-021-1094-8 34382149

[44] Homayouni A Bagheri N Mohammad-Alizadeh-Charandabi S. Prevention of gestational diabetes mellitus (GDM) and probiotics: mechanism of action: a review. Curr Diabetes Rev. 2020;16:538–45. 31544699 10.2174/1573399815666190712193828

[45] Barrett HL Callaway LK Nitert MD. Probiotics: a potential role in the prevention of gestational diabetes? Acta Diabetol. 2012;49:1–13. 23180045 10.1007/s00592-012-0444-8

[46] Chen Y Yue R Zhang B Li Z Shui J Huang X. Effects of probiotics on blood glucose, biomarkers of inflammation and oxidative stress in pregnant women with gestational diabetes mellitus: a meta-analysis of randomized controlled trials. Med Clin. 2020;154:199–206. 10.1016/j.medcli.2019.05.041 31630848

[47] Memarrast F Ghafouri‐Fard S Kolivand S. Comparative evaluation of probiotics effects on plasma glucose, lipid, and insulin levels in streptozotocin‐induced diabetic rats. Diabetes Metab Res Rev. 2017;33:e2912. 10.1002/dmrr.2912 28608654

[48] Wan J Ma J. Efficacy of dietary supplements targeting gut microbiota in the prevention and treatment of gestational diabetes mellitus. Front Microbiol. 2022;13:927883. 35910625 10.3389/fmicb.2022.927883 PMC9330481

[49] Mosavat M Omar SZ Jamalpour S Tan PC. Serum Glucose‐Dependent Insulinotropic Polypeptide (GIP) and Glucagon‐Like Peptide‐1 (GLP‐1) in association with the risk of gestational diabetes: a prospective case–control study. J Diabetes Res. 2020;2020:9072492. 32090124 10.1155/2020/9072492 PMC7008251

[50] Arain H Patel T Mureanu N. Regulatory T cells in the peripheral blood of women with gestational diabetes: a systematic review and meta-analysis. Front Immunol. 2023;14:1226617. 38111588 10.3389/fimmu.2023.1226617 PMC10726109

